# Effects of Periodic Short-Term Heat Stress on Biological Characteristics and Gut Bacteria of *Spodoptera frugiperda*

**DOI:** 10.3390/insects16060584

**Published:** 2025-06-01

**Authors:** Jingjing Jia, Min Liang, Zhitao Zhao, Weikang Huang, Qing Feng, Zhufeng Lin, Xuncong Ji

**Affiliations:** 1 Institute of Plant Protection, Research Center of Quality Safety and Standards for Agro-Products, Hainan Academy of Agricultural Sciences, Haikou 571100, China; j9405136318@163.com (J.J.);; 2 Hainan Key Laboratory for Control of Plant Diseases and Insect Pests, Haikou 571100, China; 3 College of Tropical Crops, Yunnan Agricultural University, Puer 665099, China

**Keywords:** *Spodoptera frugiperda*, periodic heat stress, biological characteristics, gut bacteria

## Abstract

In this study, the migratory agricultural pest *Spodoptera frugiperda* was exposed to three periodic short-term heat stress regimes at 37 °C, 40 °C, and 43 °C (2 h daily), with a constant 26 °C control. We systematically evaluated the effects of periodic thermal stress on developmental traits across all life stages. Combined with 16S rRNA high-throughput sequencing, we analyzed the structural and functional characteristics of the gut bacterial community in adults under heat stress. The results demonstrated that 37 °C exposure accelerated egg-to-adult development, whereas 43 °C markedly extended it. Additionally, 43 °C heat stress suppressed pupation and eclosion rates. Increasing stress temperatures were negatively correlated with pupal weight and body size in both sexes. Notably, 43 °C heat stress caused complete loss of hatching ability in offspring eggs, thereby rendering population reproduction unattainable. 16S rRNA sequencing revealed that Proteobacteria (>90%) dominated the gut bacterial community at the phylum level across all treatments. Under 43 °C heat stress, although female and male adults exhibited an increase in specific bacterial species within their gut bacteria, Alpha diversity analysis revealed no significant differences in the diversity (Shannon index) and richness (Chao index) of gut bacterial communities between sexes under temperature treatments. PICRUSt2 functional prediction indicated that metabolic pathways, biosynthesis of secondary metabolites, and microbial metabolism in diverse environments constituted the dominant functions of gut bacteria in both sexes, while heat stress exerted minimal effects on the functional profiles of gut bacteria in *S. frugiperda*. These findings not only provide a theoretical basis for predicting summer population dynamics and formulating ecological control strategies for *S. frugiperda* but also offer critical insights into the adaptive interactions between this pest and its gut bacterial community under heat stress. The results lay a foundation for further exploring the interactions between insect environmental adaptability and bacterial symbiosis.

## Simple Summary

*Spodoptera frugiperda*, a major migratory agricultural invasive pest, poses a severe threat to global food security. Owing to its unique climatic conditions, Hainan Island has become a year-round breeding ground for this pest. By employing heat shock treatments to simulate summer heat stress, this study systematically evaluated the adaptability of *S. frugiperda* to periodic short-term heat stress and characterized the changes in its biological characteristics under such conditions. Additionally, 16S rRNA high-throughput sequencing was employed to investigate the effects of such stress on the structure and function of the gut bacterial community in *S. frugiperda.* The findings not only provide critical data support for developing precise forecasting and effective control strategies for *S. frugiperda* but also lay a theoretical foundation for revealing the regulatory mechanisms of insect–microbe interactions in adapting to extreme environments from a microbial perspective.

## 1. Introduction

*Spodoptera frugiperda* (J. E. Smith), commonly known as the fall armyworm, is native to North and South America and belongs to the order Lepidoptera and family Noctuidae. As a major migratory agricultural invasive pest, it has emerged as a primary threat to global food security due to its wide host range, high fecundity, rapid migratory capability, and high potential for damaging crops [1,2,3]. In April 2019, it was first detected in Hainan Province, China. It rapidly spread across all 18 cities and counties in the province, posing a severe threat to the local maize industry and the Nanfan national breeding base. Notably, as a tropical island province with a year-round warm and humid climate, Hainan has become a permanent breeding ground for this invasive pest. However, frequent extreme high-temperature events in summer (with maximum temperatures exceeding 40 °C for multiple consecutive days) subject *S. frugiperda* to periodic short-term heat stress, presenting new challenges to its adaptive capabilities.

Temperature is a pivotal ecological factor regulating insect growth and development. Existing studies indicate that all life stages of *S. frugiperda* can develop normally at constant temperatures between 17 and 34 °C, with the optimum temperature for egg-to-adult development ranging from 26 to 30 °C [4,5,6]. However, high temperatures inhibit its reproductive capacity. When temperatures reach 32 °C, although adults remain capable of oviposition, the proportion of non-viable eggs increases significantly. At 35 °C, adult oviposition becomes abnormal, and complete reproductive arrest occurs at 37 °C [4,7]. Additionally, while the survival rate of the *S. frugiperda* population is relatively high under constant temperature conditions, natural fluctuating temperature conditions are more conducive to shortening the generation cycle, enhancing fecundity, and increasing the female proportion [8]. The rise in global average temperature has led to increases in the intensity, frequency, and duration of extreme heat events. Previous studies on climate warming and insect populations have predominantly focused on mean temperature changes, often overestimating the positive effects of temperature increases while overlooking the adverse impacts of naturally occurring extreme heat [9]. Given this, previous studies have applied short-term heat stress treatments to specific instars of *S. frugiperda,* revealing inhibitory effects of transient high temperatures across developmental stages. For the egg stage, hatching rates significantly decreased after treatment at 38 °C for 6 h or 46 °C for 2 h or longer; a 6 h treatment at 46 °C reduced hatching rates to 12.21% [10]. Further research has shown that eggs completely lose their hatching capacity when exposed to temperatures exceeding 40 °C for 3 h [11]. The tolerance of larvae to heat stress exhibits stage specificity: treatment of 1st–5th instar larvae at 43 °C for 4 h had no significant effect on the eclosion rate, while the same treatment significantly reduced the eclosion rate in 6th instar larvae [12]. The pupal stage is especially sensitive to high temperatures; a 3 h treatment at 45 °C led to 0% eclosion success [11], and although low eclosion success occurred after exposure of pupae to 46 °C for 2 h or longer, the emerged adults exhibited complete reproductive failure [10]. Ault females ceased oviposition after being exposed to 38 °C for 4 days [13]. These findings indicate that short-term heat stress severely impairs the development and reproduction of *S. frugiperda.*

In recent years, insect–microbe interactions have garnered significant research attention for their regulatory role in high-temperature adaptation. The insect gut harbors vast and diverse microbial communities, which are now recognized as a specialized “multifunctional organ” that profoundly influences insect growth and development, reproduction, nutrient metabolism, physiological activities, and immune function [14,15]. For instance, in the larvae of *Bactrocera dorsalis* and *Plagiodera versicolora*, gut bacteria promote growth and development [16,17]. In *Riptortus pedestris*, the gut bacteria *Burkholderia* are essential for host reproductive success [18]. The core gut microbes of black soldier fly larvae (*Hermetia illucens* L.) aid in protein degradation [19]. When insects are subjected to environmental stress, some symbiotic microorganisms exhibit lower high-temperature tolerance than the insects themselves, decreasing or disappearing under heat stress, thereby reducing insect adaptability. However, microorganisms in some insects can enhance host thermotolerance under high temperatures, helping hosts survive high-temperature environments [20]. Gut bacteria exhibit dual effects on hosts; on the one hand, they can help hosts adapt to the environment and maintain reproductive capacity; on the other hand, pathogenic bacteria may compromise reproductive capacity by infecting the reproductive system [21]. Studies have shown that the gut bacteria of *Eremias multiocellata* help ectotherms cope with climate warming by enhancing host immune responses [22]. Under heat stress, *Glyphodes pyloalis* improves adaptability by adjusting gut bacteria diversity and community structure [23]. Compared with their germ-free counterparts, normal *Drosophila subobscura* exhibit higher thermotolerance at 35 °C [24]. Conversely, after prolonged heat stress, the obligate symbionts *Portiera* and *Hamiltonella* in *Bemisia tabaci* are nearly eradicated, preventing their transmission to offspring [25]. These findings indicate that insect gut bacteria are closely linked to host survival, and climate warming may drive insect population declines by disrupting gut microbiota homeostasis [26], revealing complex ecological strategies of insect–microbe co-adaptation to high temperatures.

Currently, although existing studies have revealed the direct effects of short-term high temperatures on specific developmental stages of *S. frugiperda*, insects in natural environments are inevitably affected by periodic and recurring high-temperature weather changes [27]. However, the impact of periodic short-term heat stress on the population adaptation of *S. frugiperda* remains poorly understood and lacks systematic research. This study aims to simulate periodic short-term high-temperature environments to investigate their effects on the biological characteristics of *S. frugiperda*, clarify its adaptability to such stress, and provide critical biological insights for predicting and managing this pest under climate change. Additionally, using 16S rRNA high-throughput sequencing, we compare gut bacterial community structures in adult *S. frugiperda* under heat stress, establishing for the first time a direct link between gut bacterial composition and thermotolerance in this species. This research provides a mechanistic foundation for understanding the role of microorganisms in *S. frugiperda*’s response to high-temperature environments.

## 2. Materials and Methods

### 2.1. Insect Rearing

The S. frugiperda used in this study were collected from Daoyu Village, Xiuying District, Haikou City, Hainan Province, China (19°52′22.47″ N, 110°9′56.48″ E). Specimens were transported to the laboratory for multi-generational breeding and are currently maintained at the Institute of Plant Protection, Hainan Academy of Agricultural Sciences. The larvae were reared on maize leaves (variety: Meiyutian 007) in a climate chamber (Model: SAFE-RGQHS-2, Ningbo Saifu Experimental Instrument Co., Ltd., Ningbo, China) under controlled conditions of 26 ± 1 °C, 70 ± 10% relative humidity (RH), and a 12 h light:12 h dark (12L:12D) photoperiod. Additionally, adults were provided with daily nutritional supplements consisting of 10% honey water.

### 2.2. Experimental Method

#### 2.2.1. Effects of Periodic Short-Term Heat Stress on the Biological Characteristics of *Spodoptera frugiperda*

Observation of developmental duration: Newly laid egg masses of *S. frugiperda* were placed in an intelligent artificial climate chamber (rearing conditions: 26 ± 1 °C, 70 ± 10% RH, 12L:12D photoperiod). Subsequently, egg masses selected for heat treatment were exposed daily to scheduled heat stress at 37 °C, 40 °C, and 43 °C in artificial climate chambers (Model: MGC-350HP-2L, Shanghai Yiheng Scientific Instruments Co., Ltd., Shanghai, China; temperature control accuracy ±1 °C, relative humidity 70 ± 5%) for 2 h. (As newly laid egg masses failed to hatch after 2 h of treatment at 43 °C, heat stress treatments at different temperatures were initiated from the second day for egg masses). After each heat treatment, egg masses were returned to the climate chamber and reared on fresh maize leaves. Untreated egg masses served as the control group. Upon hatching, larvae were individually numbered and reared in Petri dishes (d = 10 cm). Molting and survival were recorded twice daily at 08:00 and 20:00. When larvae entered the prepupal stage, multi-layered toilet paper was provided as a pupation substrate, and pupation rates were calculated. After *S. frugiperda pupation*, daily observation was conducted until adult eclosion. Upon completion of eclosion, the sex of each numbered adult was recorded, and the egg-to-adult developmental duration was calculated separately for female and male individuals. A total of 100 individuals per treatment group were observed.

Pupal weight, eclosion rate, body length, and ovarian length measurements: Due to the large number of insects required for this experiment, *S. frugiperda* individuals not used in larval development and survival observations were subjected to the same heat stress treatments as described earlier. Eggs were placed in plastic rearing boxes (17 cm long, 15 cm wide, and 8 cm high), and larvae hatched from these eggs were fed fresh maize leaves daily. On the third day after pupation, 200 pupae were selected for weighing, and each pupa was transferred to an individual Petri dish (d = 10 cm). After all adults had eclosed, eclosion rates were calculated, and adult sex was determined. On the third day of adulthood, the body lengths of female and male adults were measured using a Keyence VHX-7000 digital microscope (Keyence Corporation, Osaka, Japan). Female adults exposed to different heat stress regimes were dissected to measure ovarian length. A minimum of 50 individuals were measured for body length (both sexes) and ovarian length (females only).

Observations of adult mating behavior: Newly eclosed *S. frugiperda* male and female adults were paired at a 1:1 ratio and individually housed in rearing boxes (upper diameter 12 cm, lower diameter 7 cm, height 6 cm), with 10% honey water provided daily as a nutritional supplement. Each pair was continuously recorded using a Xiaomi Smart Camera 3 Pro (Model: MJSXJ15CM, Shanghai Chuangmi Shulian Intelligent Technology Development Co., Ltd., Shanghai, China) for 5 consecutive days. Mating frequency, total mating duration, and mating rate were quantified. Ten pairs were observed per trial, with three independent replicates conducted.

Adult fecundity, offspring hatching rate, and longevity assessments: Newly eclosed male and female moths were paired immediately after eclosion and placed in identical rearing boxes (dimensions as above), with 10% honey water replenished daily. If a male died during the trial, it was promptly replaced with a male eclosed on the same day to maintain pairing consistency. Egg masses produced under different heat stress treatments were collected daily at 08:00. Daily fecundity (number of eggs laid) and egg hatching rate were recorded daily, continuing until adult death. Longevity of both sexes was calculated, with a minimum of 30 individuals monitored per treatment group.

#### 2.2.2. Gut Sample Collection from Spodoptera frugiperda Exposed to Periodic Short-Term Heat Stress

On the third day after eclosion, female and male *S. frugiperda* adults were selected and surface-sterilized with 75% ethanol for 3 min, followed by three rinses with phosphate-buffered saline (PBS, pH 7.2–7.4) for 1 min each. Adults were then placed in sterile Petri dishes, and intestinal tissues were dissected under a stereomicroscope. Collected gut tissues were immediately flash-frozen in liquid nitrogen and stored at −80 °C until further analysis. Thirty female adults and thirty male adults per treatment group were dissected, with five biological replicates per group.

#### 2.2.3. Gut Sample DNA Extraction, PCR Amplification, and Sequencing Library Construction

Microbial community genomic DNA was extracted from gut samples using the E.Z.N.A.^®^ Soil DNA Kit (Omega Bio-tek, Norcross, GA, USA) according to the manufacturer’s instructions. The DNA extract was checked on a 1% agarose gel, and DNA concentration and purity were determined using a NanoDrop 2000UV-Vis spectrophotometer (Thermo Scientific, Wilmington, NC, USA). For the bacterial community, the bacterial 16S rRNA genes were amplified using the universal bacterial primers 27F (5′-AGRGTTYGATYMTGGCTCAG-3′) and 1492R (5′-RGYTACCTTGTTACGACTT-3′) [28]. Primers were tailed with PacBio barcode sequences to distinguish each sample. Amplification reactions (20 μL volume) consisted of 5 × FastPfu buffer 4 μL, 2.5 mM dNTPs 2 μL, forward primer (5 μM) 0.8 μL, reverse primer (5 μM) 0.8 μL, FastPfu DNA Polymerase 0.4 μL, template DNA 10 ng, and DNase-free water. The PCR amplification was performed as follows: initial denaturation at 95 °C for 3 min, followed by 27 cycles of denaturing at 95 °C for 30 s, annealing at 60 °C for 30 s and extension at 72 °C for 45 s, and single extension at 72 °C for 10 min, and end at 4 °C (ABI GeneAmp^®^ 9700 PCR thermocyclerm, Foster, CA, USA). PCR reactions were performed in triplicate. After electrophoresis, the PCR products were purified using the AMPure^®^ PB Beads (Pacific Biosciences, Menlo Park, CA, USA) and quantified using a Quantus^TM^ Fluorometer (Promega, Madison, WI, USA). Purified products were pooled in equimolar amounts, and a DNA library was constructed using the SMRTbell^®^ Express Template Prep Kit 2.0 (Pacific Biosciences, Menlo Park, CA, USA) according to PacBio’s instructions. Purified SMRTbell libraries were sequenced on the PacBio Sequel II System (Pacific Biosciences, Menlo Park, CA, USA) by Majorbio Bio-Pharm Technology Co., Ltd. (Shanghai, China).

### 2.3. Data Processing and Statistical Analysis

PacBio raw reads were processed using the SMRT Link analysis software (v8.0) to obtain demultiplexed circular consensus sequence (CCS) reads with a minimum of 3 full passes and 99% sequence accuracy. CCS reads were barcode-identified and length-filtered. For the bacterial 16S rRNA gene, sequences with a length <1000 or >1800 bp were removed. The optimized-CCS reads were clustered into operational taxonomic units (OTUs) using UPARSE v7.1 [29,30] with a 97% sequence similarity level. The taxonomy of each OTU representative sequence was analyzed by RDP Classifier v 2.2 [31] against the 16S rRNA gene database (Silva v138) using a confidence threshold of 0.7. The metagenomic function was predicted by PICRUSt2 (Phylogenetic Investigation of Communities by Reconstruction of Unobserved States) [32] based on OTU representative sequences. Bioinformatics analysis of the gut microbiota was carried out using the Majorbio Cloud platform (https://cloud.majorbio.com). Alpha diversity metrics, such as the Chao index and Shannon index, were calculated using Mothur v1.30.1 [33]. The similarity among the microbial communities in different samples was determined by principal component analysis (PCA) based on Euclidean distance using the Vegan v2.5-3 package. The PERMANOVA test was used to assess the percentage of variation explained by the treatment, along with its statistical significance using the Vegan v2.5-3 package.

Statistical analysis of biological data for *S. frugiperda* was conducted as follows: Normality tests were performed using SPSS v20.0 (IBM, Armonk, NY, USA). For normally distributed data, one-way ANOVA was performed to assess intergroup differences, followed by Duncan’s multiple range test for post hoc comparisons. Results were visualized as bar charts showing mean ± standard error (SE). Non-normally distributed data were analyzed using the Kruskal–Wallis test in GraphPad Prism v10.0 (GraphPad Software, San Diego, CA, USA), followed by Dunn’s post hoc test. Results were presented as box plots showing median and interquartile range (IQR). A mixed-effects model was applied to developmental time data across stages, incorporating the interaction between stress temperature and developmental time. Simple effects tests were then performed, with *p*-values corrected using Tukey’s method. All visualizations were generated in GraphPad Prism, and image post-processing was performed using Adobe Photoshop 2022 (Adobe, San Jose, CA, USA).

## 3. Results

### 3.1. Effects of Periodic Short-Term Heat Stress on the Developmental Duration of Spodoptera frugiperda

**Figure 1:**
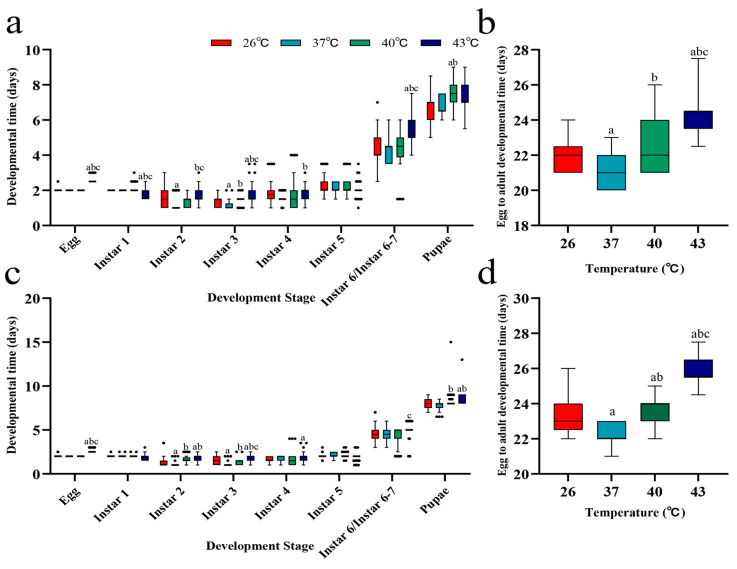
Developmental duration of *S. frugiperda* under periodic short-term heat stress. (**a**) Developmental time of different stages in female insects. (**b**) Egg-to-adult developmental duration in females. (**c**) Developmental time of different stages in male insects. (**d**) Egg-to-adult developmental duration in males. a indicates *p* < 0.05 compared with 26 °C; b indicates *p* < 0.05 compared with 37 °C; c indicates *p* < 0.05 compared with 40 °C.

Under periodic short-term heat stress at 37 °C and 40 °C, the hatching time of *S. frugiperda* eggs showed no significant difference compared with the 26 °C control group (*p* > 0.05). However, at 43 °C, hatching time was significantly prolonged compared to other temperature treatments (*p* < 0.05) (Figure 1a,c). Further observations revealed that the shortest egg-to-adult developmental duration occurred under 37 °C stress, whereas the 43 °C stress extended developmental duration. These results indicate that moderate short-term heat stress promotes *S. frugiperda* development, but temperatures exceeding a critical threshold delay it instead (Figure 1b,d).

### 3.2. Effects of Periodic Short-Term Heat Stress on Larval Survival Rate, Pupation Rate, and Eclosion Rate of Spodoptera frugiperda

**Figure 2:**
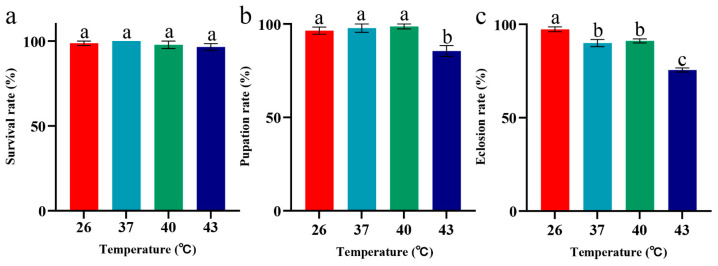
Larval survival rate, pupation rate, and eclosion rates of *S. frugiperda* under periodic short-term heat stress. (**a**) Survival rate of first-instar to last-instar larvae. (**b**) Larval pupation rate. (**c**) Adult eclosion rate. The data in the figure are mean ± SE. Different lowercase letters above bars indicate significant differences (*p* < 0.05).

Under periodic heat stress at 37 °C, 40 °C, and 43 °C, *S. frugiperda*’s larval survival rates showed no significant difference from the 26 °C control group (*p* > 0.05). Even under 43 °C stress, where the larval survival rate was the lowest, the rate remained above 96%, indicating strong tolerance of the larval stage to periodic short-term heat stress (Figure 2a). However, both pupation and adult eclosion rates of *S. frugiperda* subjected to stress at 43 °C were significantly lower than those in other temperature groups (*p* < 0.05), thus suggesting that pupal stage development is sensitive to extremely high temperatures (Figure 2b,c).

### 3.3. Effects of Periodic Short-Term Heat Stress on Pupal Weight, Adult Body Length, and Ovarian Length of Spodoptera frugiperda

**Figure 3:**
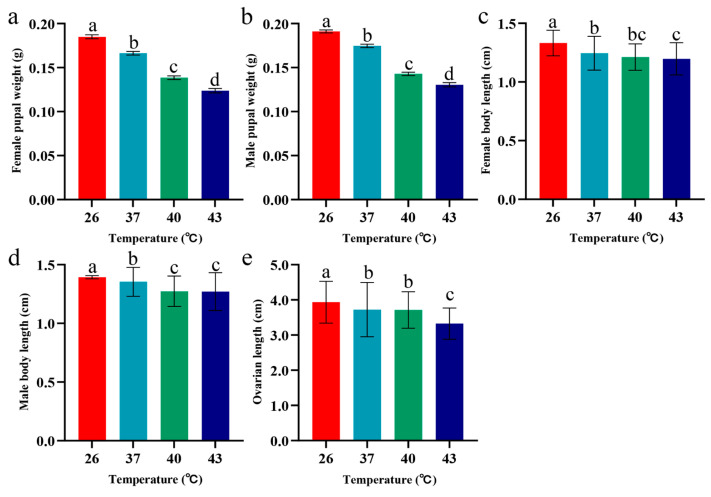
Pupal weight, adult body length, and ovarian length of *S. frugiperda* under periodic short-term heat stress. The data in the figure are mean ± SE. Different lowercase letters above bars indicate significant differences (*p* < 0.05). (**a**) Female pupal weight. (**b**) Male pupal weight. (**c**) Female body length. (**d**) Male body length. (**e**) Ovarian length.

Periodic short-term heat stress had significantly different effects on female and male pupal weights of *S. frugiperda* (*p* < 0.05). As the stress temperature increased, both female and male pupal weights exhibited a significant downward trend; the weights of female and male pupae in the 43 °C stress group were 33.14% and 31.71% lower, respectively, than those in the 26 °C control group (Figure 3a,b). Additionally, heat stress inhibited adult body size development, with the body lengths of both female and male adults decreasing linearly as the temperature increased. The body lengths of female and male adults in the 43 °C stress group were 9.44% and 8.81% shorter, respectively, than those in the 26 °C control group (Figure 3c,d). Heat stress impacted ovarian development, with ovarian length of female adults decreasing as stress temperature increased. Specifically, the ovarian length in the 43 °C stress group was 15.46% shorter than that in the control group (Figure 3e).

### 3.4. Effects of Periodic Short-Term Heat Stress on Mating of Spodoptera frugiperda

**Figure 4:**
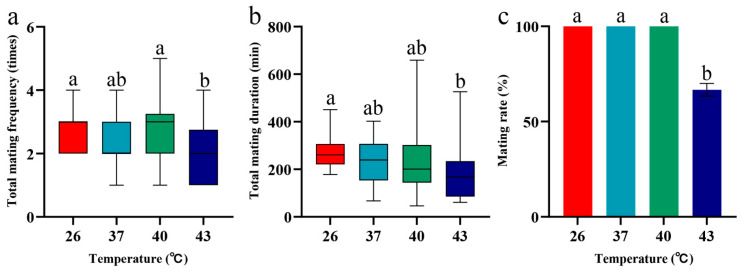
Mating behavior of *S. frugiperda* under periodic short-term heat stress. Box plots show the median and interquartile range (IQR); bar charts present data as mean ± SE. Different lowercase letters above the boxplots and bar charts indicate significant differences (*p* < 0.05). (**a**) Total mating frequency. (**b**) Total mating duration. (**c**) Mating rate of female and male adults.

After periodic short-term heat stress at 37 °C and 40 °C, the adult mating frequency, mating duration, and mating success rate of *S. frugiperda* showed no significant differences compared with the 26 °C control group (*p* > 0.05), with mating success rates all reaching 100% (Figure 4). However, following periodic short-term heat stress at 43 °C, both the mating frequency and mating success rate of *S. frugiperda* were significantly lower than those in the 26 °C control group (*p* < 0.05) (Figure 4a,c).

### 3.5. Effects of Periodic Short-Term Heat Stress on Adult Fecundity and Longevity of Spodoptera frugiperda

**Figure 5:**
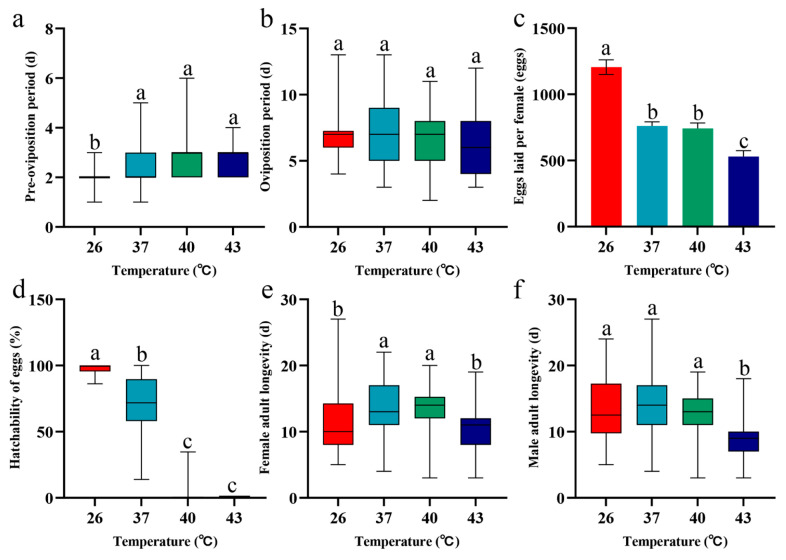
Fecundity and longevity of adult *S. frugiperda* under periodic short-term heat stress. Box plots show the median and interquartile range (IQR); bar charts present data as mean ± SE. Different lowercase letters above the boxplots and bar charts letters indicate significant differences (*p* < 0.05). (**a**) Pre-oviposition period. (**b**) Oviposition period. (**c**) Eggs laid perf female. (**d**) Hatchability of eggs. (**e**) Female adult longevity. (**f**) Male adult longevity.

**Figure 6:**
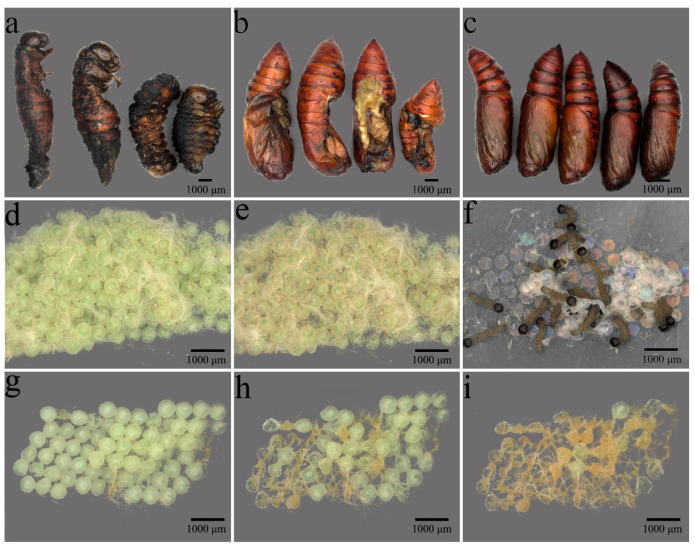
Effects of periodic short-term heat stress on pupation and offspring egg hatching in *S. frugiperda*. (**a**,**b**) Individuals with abnormal pupation. (**c**) Individuals with normal pupation. (**d**–**f**) Days 1–3 of offspring egg development under 26 °C. (**g**–**i**) Days 1–3 of offspring egg development under 43 °C stress treatment.

After periodic short-term heat stress at different temperatures, the pre-oviposition period of *S. frugiperda* adults was significantly prolonged compared to the 26 °C control group (*p* < 0.05) (Figure 5a), whereas no significant differences in oviposition period were observed among temperature treatments (*p* > 0.05) (Figure 5b). Meanwhile, both the number of eggs laid per female and the offspring egg hatching rate decreased as stress temperature increased. Compared to the 26 °C control group, the number of eggs laid per female in the 37 °C, 40 °C, and 43 °C stress groups decreased by 36.88%, 38.45%, and 56.12%, respectively. At 40 °C stress, the egg hatching rate of the offspring was only 2.60%, and no eggs hatched when the stress temperature increased to 43 °C (Figure 5d, Figure 6i). Additionally, the responses of adult lifespan to heat stress exhibited sex- and temperature-dependent variations; following periodic short-term heat stress at 37 °C and 40 °C, female adult longevity was significantly longer than that of the 26 °C control group (*p* < 0.05) (Figure 5e). Male adult longevity under 37 °C stress was also longer than that of the control group, though not significantly (*p* > 0.05). Heat stress at 43 °C shortened the lifespan of female and male adults by 7.97% and 36.88%, respectively, compared to the 26 °C control group (Figure 5e,f).

### 3.6. Analysis of Gut Bacterial Community Sequencing Data and Species Composition in Female and Male Adults of Spodoptera frugiperda Under Periodic Short-Term Heat Stress

A total of 40 samples from male and female adults of *S. frugiperda* under different temperatures were subjected to gut bacteria sequencing analysis, yielding 1,772,102 quality-filtered sequences spanning 2,565,208,872 bases with an average length of 1447 bp. Taxonomic annotation identified nine phyla, 16 classes, 30 orders, 61 families, 103 genera, and 131 species.

**Figure 7:**
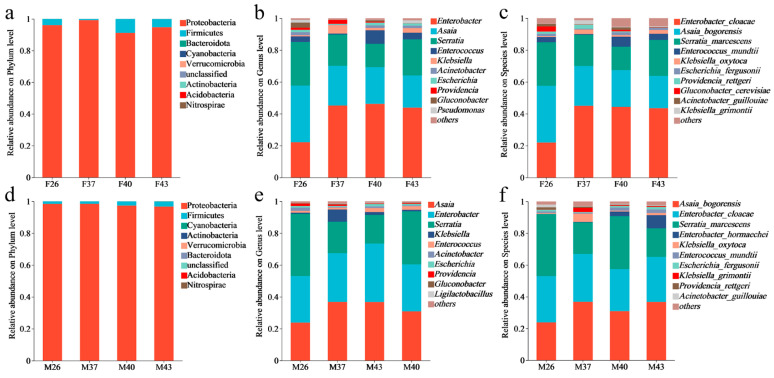
Bar plots of the gut bacterial communities structure in female and male adults of *S. frugiperda* under different periodic short-term heat stress. (**a**–**c**) Relative abundances of female adults’ gut bacteria at the phylum, genus, and species levels, respectively; (**d**–**f**) relative abundances of male adults’ gut bacteria at the phylum, genus, and species levels, respectively. F: female adults; M: male adults; values: treatment temperatures (°C). The same notation applies to subsequent figures.

At the phylum level, the dominant gut bacteria in both female and male adults of *S. frugiperda* under different temperature stress were Proteobacteria, with relative abundances exceeding 90% in all groups. At the genus level, female and male adults shared similar dominant genera, with the top three most abundant genera being *Asaia*, *Enterobacter*, and *Serratia* (Figure 7).

**Figure 8:**
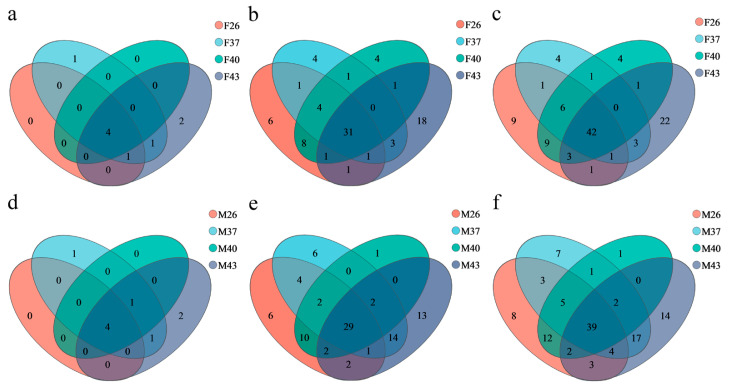
Venn diagrams of the gut bacterial communities composition in female and male adults of *S. frugiperda* under different periodic short-term heat stress. (**a**–**c**) Shared and unique taxa in the gut bacterial communities of female adults at the phylum, genus, and species levels, respectively. (**d**–**f**) Shared and unique taxa in the gut bacterial communities of male adults at the phylum, genus, and species levels, respectively.

Under different temperature stress treatments, the gut bacterial community of adult female *S. frugiperda* consisted of 42 species, classified into four phyla and 31 genera, whereas that of male adults shared 39 bacterial species, classified into four phyla and 29 genera (Figure 8). Analysis of species-level unique bacteria revealed that under the 26 °C control condition, females harbored nine unique gut bacterial species, while males had eight. After short-term heat stress at 37 °C and 40 °C, the number of unique species in females remained at four in both treatments, whereas in males it decreased to seven (37 °C) and one (40 °C), respectively. Under extreme heat stress at 43 °C, the number of unique species in females increased to 22 and in males to 14, both significantly higher than in other treatments. These results indicate that periodic short–term heat stress at 43 °C significantly induced the enrichment of unique gut bacterial species in both sexes, with females exhibiting a more pronounced response (Figure 8c,f).

### 3.7. Analysis of Gut Bacterial Communities Diversity and Richness in Female and Male Adults of Spodoptera frugiperda Under Periodic Short-Term Heat Stress

**Figure 9:**
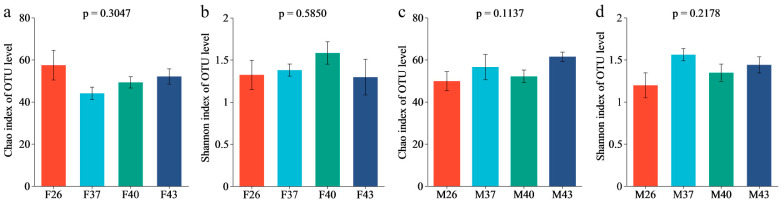
Alpha diversity analysis of the gut bacterial communities in female and male adults of *S. frugiperda* under different periodic short-term heat stress. (**a**) Female Chao index. (**b**) Female Shannon index. (**c**) Male Chao index. (**d**) Male Shannon index. The data in the figure are mean ± SE. Statistically significant differences (*p* < 0.05) indicate marked variations in the corresponding indices among temperature groups.

Alpha diversity analysis showed that periodic short–term heat stress had no significant effects on the diversity and richness of gut bacterial communities in female and male adults of *S. frugiperda* (*p* > 0.05) (Figure 9).

**Figure 10:**
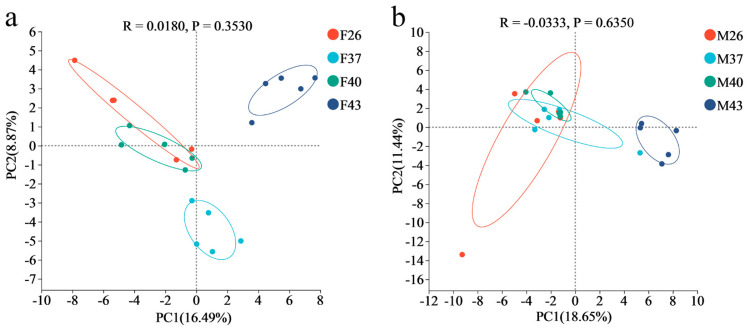
PCA analysis of the gut bacterial communities in female and male adults of *S. frugiperda* under periodic short-term heat stress. (**a**) PCA of female gut bacterial communities. (**b**) PCA of male gut bacterial communities. PC1 and PC2 represent the first two principal components, with percentages indicating the proportion of variance explained by each component in compositional differences among samples. Points of different colors correspond to distinct sample groups; closer proximity between points reflects higher similarity in species composition.

Principal component analysis (PCA) revealed that the gut bacterial communities of female *S. frugiperda* adults in the 26 °C control group exhibited high similarity to those exposed to periodic 40 °C heat stress, whereas females under 43 °C stress showed distinct separation from other temperature treatments. In male adults, the bacterial composition of the 26 °C control group closely resembled that under 37 °C and 40 °C stress. Although clear differentiation was observed between the 26 °C control and 43 °C stress groups, no significant differences in gut bacterial community structure were detected between female and male adults across treatments (Figure 10).

### 3.8. Differential Analysis of Gut Bacterial Composition in Female and Male Adults of Spodoptera frugiperda Under Periodic Short-Term Heat Stress

**Figure 11:**
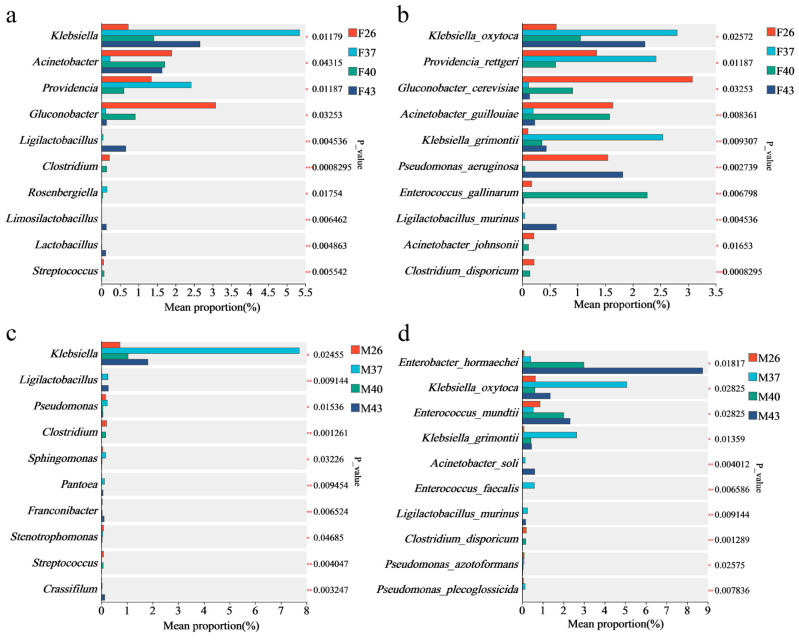
Differential analysis of gut bacteria in female and male adults of *S. frugiperda* under periodic short-term heat stress. (**a**) Genus-level differences in female gut bacteria. (**b**) Species-level differences in female gut bacteria. (**c**) Genus-level differences in male gut bacteria. (**d**) Species-level differences in male gut bacteria. Asterisks indicate significance levels: * *p* ≤ 0.05, ** *p* ≤ 0.01, and *** *p* ≤ 0.001.

At the genus level, compared with the 26 °C control group, periodic short-term heat stress at 43 °C resulted in the absence of *Providencia*, *Clostridium*, and *Streptococcus* in the gut of female adults, while *Ligilactobacillus*, *Limosilactobacillus*, and *Lactobacillus* were enriched (Figure 11a). In male adults, 43 °C short-term heat stress led to the absence of *Clostridium*, *Sphingomonas*, *Stenotrophomonas*, and *Streptococcus,* with significant enrichment of *Ligilactobacillus*, *Pantoea*, and *Crassifilum.*

At the species level, compared with the 26 °C control group, the intestinal bacteria of female adults exhibited increased abundances of *Klebsiella oxytoca* and *Klebsiella grimontii* after 37 °C, 40 °C, and 43 °C heat stress. Specifically, *K. oxytoca* increased by 354.03%, 2334.87%, and 70.91% in the 37 °C, 40 °C, and 43 °C stress groups, respectively, while *K. grimontii* showed increases of 259.8%, 241.86%, and 316.86%. Notably, *Pseudomonas aeruginosa* and *Enterococcus gallinarum* were completely absent after 37 °C stress, whereas *Acinetobacter soli* and *Ligilactobacillus murinus* were detected. Under 43 °C stress, *Providencia rettgeri* disappeared, but *L. murinus* remained detectable (Figure 11b). In male adults, the abundance of *Enterobacter hormaechei* increased progressively with rising stress temperatures, showing 342.75%, 3099.36%, and 9239.02% increases in the 37 °C, 40 °C, and 43 °C groups compared to the 26 °C control. Similar to females, the abundance of *K. grimontii* also increased across stress groups, with increments of 2830.00%, 374.56%, and 402.22% at 37 °C, 40 °C, and 43 °C, respectively. Strikingly, *A. soli* and *L. murinus* were exclusively observed in the 37 °C and 43 °C stress groups (Figure 11d).

### 3.9. Functional Prediction of Gut Bacterial Communities in Female and Male Adults of Spodoptera frugiperda Under Periodic Short-Term Heat Stress

**Figure 12:**
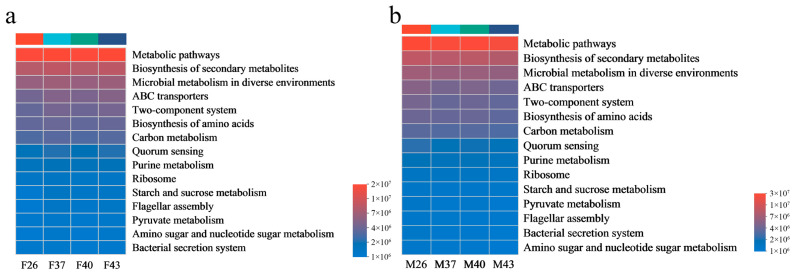
Functional potential prediction (PICRUSt2) of the 15 most abundant gut bacterial taxa in adult females and males of *S. frugiperda* under periodic short-term heat stress. (**a**) Female gut bacterial functional profiles; (**b**) male gut bacterial functional profiles.

Functional prediction results from PICRUSt2 level 3 analysis of gut bacterial communities in female and male adults of *S. frugiperda* under periodic short-term heat stress showed that metabolic pathways—core metabolism-related functions—had the highest relative abundance in both sexes across all temperature treatments. Subsequent enriched functions included biosynthesis of secondary metabolites and microbial metabolism in diverse environments, indicating consistent functional profiles between female and male gut bacterial communities and minimal impacts of periodic short-term heat stress on gut bacterial functions (Figure 12). Further analysis revealed that in female adults, the relative abundances of ABC transporters and two-component systems were higher at 37 °C and 43 °C than at 26 °C and 40 °C (Figure 12a), whereas in male adults, these two functions exhibited opposite abundance trends (Figure 12b).

## 4. Discussion

After experiencing periodic short-term high-temperature stress above 40 °C, the development time of *S. frugiperda* is significantly prolonged. This phenomenon aligns with observations in *Myzus persicae* under 36 °C short-term heat stress, where the developmental rate gradually decelerates with prolonged exposure [34]. These results indicate that high temperatures exceeding insect tolerance thresholds—whether applied as periodic short-term or stage-specific stress—inhibit development, with the inhibitory effect intensifying as thermal exposure duration increases. Notably, interspecific disparities in thermal responses exist; while *S. frugiperda* shows developmental delay, *Ostrinia furnacalis* exhibits accelerated egg-to-larval development as temperature or treatment duration increases [35]. Such discrepancies may relate to species-specific high-temperature adaptation strategies or to differences in tolerance across developmental stages. Developmental stage analysis reveals that the larval stage exhibits stronger tolerance to short-term heat stress. In this study, we observed no significant differences in survival rates among *S. frugiperda* larvae subjected to different periodic short-term heat stresses, consistent with the finding that 3rd-instar larvae maintain 100% survival after 3 h at 40 °C and 45 °C [11]. By contrast, the pupal stage and eclosion are more sensitive; periodic short-term stress at 43 °C significantly reduces both pupation and eclosion rates. For instance, treating 3rd-instar larvae at 36 °C for 24 and 32 h decreases pupation rates by 49.8% and 65.0%, respectively [36]. Pupae are particularly vulnerable; exposure to 35–38 °C for 2–4 days reduces eclosion to 30–60%, and temperatures ≥ 41 °C cause complete pupal mortality within 2 days [13]. Treatment at 45 °C for 3 h results in 0% eclosion [11]. Collectively, these findings demonstrate stage-specific inhibitory effects of heat stress on *S. frugiperda*; short-term heat stress minimally impacts larval survival, whereas pupation and eclosion are highly sensitive to high temperatures, with inhibition intensifying as temperature and exposure duration increase.

Heat stress exerts significant impacts on insect pupal weight and body size. This study found that under periodic short-term heat stress, pupal weight of *S. frugiperda* gradually decreases with increasing temperature, and body length shortens correspondingly. This finding aligns with prior studies across diverse temperature ranges; within 19–31 °C, male pupae exhibit significantly higher weights than females [37]; within 20–34 °C, female pupal weight declines with temperature, and males maintain higher weights at all temperatures except 20 °C [38]. This sexual dimorphism likely reflects divergent energy allocation strategies; males allocate more resources to somatic growth, whereas females prioritize reproductive organ development. A parallel adaptive strategy of body size reduction has been documented in the pea aphid *Acyrthosiphon pisum* [39], suggesting convergent evolution for thermal adaptation. These findings imply that insects may reduce energy expenditure by decreasing body size to cope with high-temperature stress. Regarding adult ovarian development, this study revealed that thermal stress significantly suppresses ovarian growth in female *S. frugiperda*. As stress temperature increased, ovarian length exhibited a marked shortening trend, with significant differences between heat-stressed groups and the 26 °C control. This temperature-dependent suppression is widespread across insect taxa; in *Agasicles hygrophila*, short-term heat stress caused progressive shortening of ovarian tubules within the first 4 days, but unexpectedly induced tubule elongation by day 5, highlighting dynamic thermal impacts on reproductive development [40]. Similarly, pupal-stage heat stress in *Bradysia odoriphaga* showed negative correlations between adult ovarian length and both stress temperature and duration [41]. Notably, *Cotesia vestalis* displayed temporal plasticity in ovarian recovery; 3-day-old pupae exposed to heat stress produced adults with shortened ovarian tubules during early eclosion (days 1–5), but differences dissipated by day 7 post-eclosion [42]. Collectively, these findings demonstrate that thermal effects on insect ovarian development are characterized by pronounced temporal dynamics and species specificity, with effect magnitudes governed by the interplay of stress intensity, developmental stage sensitivity, and host repair capacity.

Temperature is a key determinant of insect mating behavior, including duration, sperm transfer, and frequency [43]. Our study revealed that periodic short-term heat stress within moderate ranges (≤40 °C) did not significantly affect *S. frugiperda* mating frequency or success rate; however, at 43 °C, both parameters decreased sharply, indicating that extreme temperatures strongly inhibit mating. This aligns with prior research showing that *Drosophila melanogaster* exhibits reduced mating rates at 33 °C compared to 25 °C, while heat-hardened individuals maintain higher mating rates at 33 °C [44]. Similarly, mating duration and frequency decline progressively with rising temperatures in *Bactrocera dorsalis* and *Anaphes listronoti* within specific thermal ranges [45,46]. These findings underscore the conserved inhibitory effects of heat stress on insect mating while highlighting interspecific variability in thermal tolerance. Notably, while periodic short-term heat stress did not alter the oviposition period of *S. frugiperda*, it significantly reduced population reproduction. As stress temperature increased, female fecundity declined, with the hatching rate being particularly sensitive; it dropped to 2.6% under 40 °C stress and reached 0% at 43 °C. These results contrast with previous reports where 2 h exposure to 43 °C on the day of eclosion resulted in a 12.6% hatching rate [47], and 5-day adult treatment at 43 °C allowed a 33.8% hatching rate [12]. This discrepancy may stem from differences in stress timing. Unlike prior studies, our periodic short-term stress began at the egg stage, potentially amplifying cumulative damage across developmental stages. The pupal phase, a critical period for tissue remodeling, may be particularly susceptible to heat-induced ovarian malformations or reduced sperm quality, ultimately impairing offspring fitness. Our results demonstrate that periodic heat stress (≥40 °C) significantly suppresses *S. frugiperda* population growth, advancing mechanistic understanding of thermal effects on insect demographics. Intriguingly, the lifespan of female adults exposed to heat stress at 37 °C and 40 °C was significantly prolonged compared to those at 26 °C. This result contrasts with previous findings showing that adult lifespan is significantly shortened after short-term heat stress during the pupal stage in *S. frugiperda* or direct exposure to 40 °C and 43 °C [12,13]. However, some studies reported no significant lifespan differences in *S. frugiperda* adults exposed to 41 °C for varying durations compared to the 25 °C control group [47]. Similar lifespan extension phenomena under heat stress have been observed in other insects; female adults of *A. hygrophila* showed no significant longevity changes after short-term exposure to 40–44 °C [48], while studies on *Grapholita molesta* demonstrated that adult lifespan increased significantly with higher temperatures (except at 38 °C), with both sexes surviving longer at 41 °C than the control group [49]. These discrepancies may be attributed to differences in stress timing (developmental stage) or species-specific energy allocation strategies. Through such strategies, adults may prioritize survival over reproduction within an optimal high-temperature range to mitigate heat stress impacts.

Our study revealed that under periodic short-term heat stress, the gut bacteria of both female and male *S. frugiperda* adults were dominated by *Proteobacteria* at the phylum level. Notably, thermal stress induced a genus-level shift in females; *Asaia*, the dominant genus under control conditions, was replaced by *Enterococcus*, suggesting enhanced heat tolerance of the latter. Despite these compositional shifts, periodic short-term heat stress did not significantly affect gut bacterial diversity or richness in either sex. Existing studies show that short-term environmental fluctuations have minimal impact on *S. frugiperda* gut bacteria, whereas long-term habitat differences can reshape community composition [50]. This stability may stem from the conserved nature of laboratory-reared populations. For instance, parasitism by *Cotesia ruficrus* fails to alter gut bacterial diversity in laboratory populations, in contrast to field populations that exhibit marked shifts [51]. Similarly, laboratory populations maintain stable microbiota even under prolonged exposure to polyethylene microplastics [52]. Parallel findings exist in other insects; *Zeugodacus cucurbitae* shows stable microbial diversity across temperatures [53], and *Propylea japonica* exhibits minimal gut bacteria compositional changes under heat stress [54]. However, most studies report temperature-dependent effects on insect gut microbiota. In *Ostrinia furnacalis*, gut bacteria diversity and abundance display a “short-term increase followed by long-term decrease” pattern with prolonged heat stress [35], highlighting stress duration as a critical factor. In *Bombyx mori*, short-term heat stress reduces genus-level microbiota abundance, with females being more sensitive [55], possibly linked to sex-specific gut microenvironmental variations. Daily 3 h 40 °C stress throughout the lifecycle of *Sarcophaga peregrina* induces significant microbial community shifts [56]. Collectively, these findings suggest that thermal impacts on gut microbiota depend on stress intensity, duration, and host developmental stage. Sex-specific analysis revealed that *Providencia* disappeared from female guts under 43 °C stress, potentially due to genus-specific host regulation. *Providencia* produces neurotransmitters mimicking host molecules, influencing sensory behavior [57]. In laboratory *Ceratitis capitata*, artificial injection of *Providencia rettgeri* increases mortality [58], implying that heat-induced *Providencia* loss may enhance host survival by reducing neural metabolic load. In males, by contrast, *E. hormaechei* abundance increased with temperature, a bacterium that maintains gut homeostasis by inhibiting pathogens [59]. Additionally, *Enterococcus mundtii*—which protects *Tribolium castaneum* against *Bacillus thuringiensis* [60]—was enriched under 43 °C stress, suggesting a role in immune defense. While these heat-responsive microbes exhibit functional roles in host physiology and microecology, their specific contributions to *S. frugiperda* heat tolerance remain unclear. Future studies should validate their functional roles, providing a comprehensive basis for understanding insect thermal adaptation mechanisms.

Based on the functional prediction analysis of PICRUSt2, this study found that metabolic functions are most abundant in the gut bacterial community of both female and male *S. frugiperda* adults under periodic short-term thermal stress. Notably, PICRUSt2-based predictions rely on phylogenetic relationships derived from 16S rDNA sequences and reference database alignments. However, these methods may introduce predictive biases or functional annotation errors due to inherent database limitations. Despite these limitations, previous studies have shown that, whether analyzed via metagenomic or 16S rDNA sequencing, *S. frugiperda* gut microbiota consistently exhibit enriched carbohydrate metabolic functions, irrespective of host plant diet or developmental stage [61,62]. Metagenomic sequencing further revealed that metabolic pathways remained the predominant functional category in *S. frugiperda* gut microbiota under graphene oxide exposure [63]. These findings collectively indicate that while external environmental factors drive gut microbiota compositional shifts, core metabolic functions exhibit remarkable stability—a likely adaptive strategy for *S. frugiperda* to thrive in dynamic environments.

## 5. Conclusions

In summary, this study represents the first investigation into the adaptability of *S. frugiperda* to periodic short-term heat stress and the response of adult gut bacteria to heat stress. The findings reveal that periodic short-term heat stress (≥40 °C) significantly impairs the reproductive capacity of *S. frugiperda*, with severity escalating as temperature increases. At 43 °C, complete reproductive failure occurred, characterized by zero viable offspring production. Analysis of adult gut microbiota showed that *Proteobacteria* predominate at the phylum level in both sexes. Notably, a thermal-induced shift in dominant genera was observed; females transitioned from *Asiaticobacter* to *Enterococcus* with rising temperatures, whereas males exhibited enrichment of *Enterobacter* and *E. mundtii*. However, short-term heat stress did not significantly alter gut bacterial α-diversity, and notably, core metabolic pathways remained stable. Against the backdrop of global warming-induced extreme heat events, these findings provide critical insights for predicting the population dynamics of this invasive pest and developing targeted management strategies. They also establish a foundation for elucidating host–microbiome interactions under thermal stress from a symbiotic perspective. Future studies could integrate bacteria functional validation with host physiological response analyses to further unravel the adaptive strategies by which *S. frugiperda* copes with extreme temperatures.

## Data Availability

Raw sequencing data were deposited in the NCBI Short Read Archive (SRA) BioProject PRJNA1256705.
